# Cost Dynamics of Clean Energy Technologies

**DOI:** 10.1007/s41471-021-00114-8

**Published:** 2021-09-07

**Authors:** Gunther Glenk, Rebecca Meier, Stefan Reichelstein

**Affiliations:** 1 Mannheim Institute for Sustainable Energy Studies, University of Mannheim, Mannheim, Germany; 2 Graduate School of Business, Stanford University, Stanford, USA

**Keywords:** Learning-by-doing, Renewable energy, Energy storage, Electrolysis, Levelized cost of energy, M1, O33, Q41, Q42, Q48, Q54, Q55

## Abstract

The pace of the global decarbonization process is widely believed to hinge on the rate of cost improvements for clean energy technologies, in particular renewable power and energy storage. This paper adopts the classical learning-by-doing framework of Wright (1936), which predicts that cost will fall as a function of the cumulative volume of past deployments. We first examine the learning curves for solar photovoltaic modules, wind turbines and electrolyzers. These estimates then become the basis for estimating the dynamics of the life-cycle cost of generating the corresponding clean energy, i.e., electricity from solar and wind power as well as hydrogen. Our calculations point to significant and sustained learning curves, which, in some contexts, predict a much more rapid cost decline than suggested by the traditional 80% learning curve. Finally, we argue that the observed learning curves for individual clean energy technologies reinforce each other in advancing the transition to a decarbonized energy economy.

**Electronic supplementary material:**

The online version of this article (10.1007/s41471-021-00114-8) contains supplementary material, which is available to authorized users.

## Introduction

A growing chorus of voices from the scientific community, policymakers, and business leaders point to climate change as an ever more urgent threat to the stability of the world’s biosphere and, hence, to economic prosperity. At the same time, the global economy has thus far failed to bend the curve of carbon dioxide (CO_2_) emissions, at least until the beginning of the Covid-19 pandemic in 2020 (Le Quéré et al. 2020). Bending this curve would be a modest first step towards staying within the remaining “carbon budget” that is viewed as being compatible with an increase in global temperature in the range of the 1.5 to 2 °C, relative to pre-industrial levels.^1^

A central issue in the debate about the timely completion of the decarbonization process is how quickly the economics of carbon-free energy technologies is improving.^2^ Some prominent observers have pointed to the need for technological innovations that represent “breakthroughs” rather than continued incremental improvements in clean energy technologies that are already mainstream (Gates 2021). In this context, the Energy Secretary in the Biden administration, Jennifer Granholm, testified to Congress in April of 2021: *“Over the coming weeks, we at the Department of Energy will be announcing new goals for bold, achievable leaps in next-generation technologies—starting with hydrogen, carbon capture, industrial fuels, and energy storage. We will marshal our National Labs, our universities, and our private sector to unlock major breakthroughs. So we’ve already announced a goal of cutting the price of solar in half yet again by 2030. And next, we’ll start lowering the cost of clean, renewable hydrogen by 80 percent before 2030, making it competitive with natural gas.”*

The analysis in this paper speaks to the prospects of meeting the 2030 goals articulated by the U.S. Department of Energy. Our approach relies on quantifying the observed past learning curves for several energy generation and storage technologies. Specifically, we compare the rate of economic progress for established clean energy technologies, including solar photovoltaic (PV) power, onshore wind power and lithium-ion batteries. Our analysis also covers the “next generation technology” of producing hydrogen through water electrolysis.

**Fig. 1:**
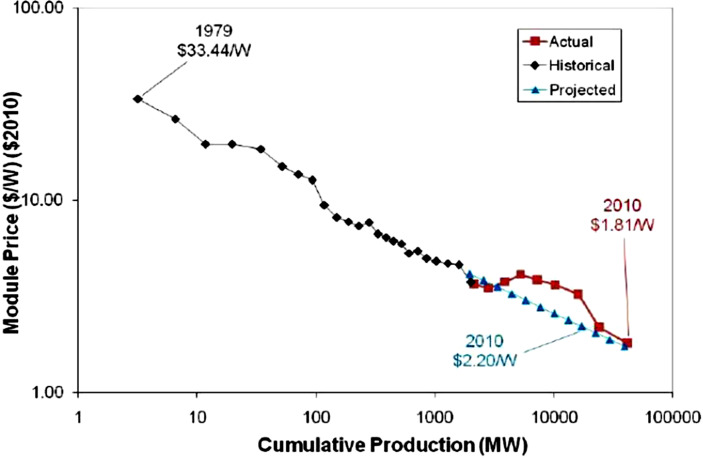
Historic solar PV module prices (Swanson 2011)

The well-known graph reproduced in Fig. 1 is commonly attributed to R. Swanson, the former CEO of the solar company SunPower. Swanson (2011) simply plotted the selling prices of solar photovoltaic PV modules, measured in 2010 dollars, per Watt of peak power against the cumulative number of solar PV modules produced since 1978. With both cumulative volume, $Q$, and prices, $P$, measured on a logarithmic scale, Fig. 1 yields a statistically near-perfect relation corresponding to a constant elasticity learning curve of the form:

1 $$P=a\cdot Q^{-b},$$

or, equivalently $\ln(P)=\ln(a)-b\cdot\ln(Q)$. The functional specification in equation (1) is frequently attributed to Wright (1936).^3^ If the slope coefficient is estimated at $b=0.322$, the corresponding prediction is that with every doubling of cumulative output the corresponding price per module is only 80% of the previous price before the doubling. This prediction reflects that $2^{-0.322}\approx 0.80$. Since a simple regression of the data points in Fig. 1 returns a $b\approx 0.322$, Fig. 1 is commonly referred to as the *80% learning curve*, with a corresponding *learning rate* of 20%.^4^

Technological progress and the main drivers underlying such progress have been examined in various models in the management and industrial organization literature (Porter 1990; Tirole 1988; Lieberman 1984). One prominent example is *Moore’s Law*, named after the former CEO of Intel Corporation, in connection with semiconductor devices.^5^ The key difference between this specification and Wright’s constant elasticity model in equation (1) is that technological progress is an exogenous function of time in Moore’s formulation, while in the Wright formulation the rate of progress is endogenous and driven by the rate of technology deployment. The implications for the clean energy transition and climate change are fundamental. In Wright’s formulation, deployments of a clean energy technology not only have an immediate effect in terms of decarbonizing a slice of the current energy system but also a future learning effect in terms of bringing down the cost of future deployments of the same technology. Our empirical estimates confine attention to the constant elasticity learning curve framework, without seeking to validate that framework relative to alternative models of technological progress.

For the energy technologies considered in this paper, we first estimate the price dynamics of key system components, e.g., the modules for solar PV power systems or the electrolyzer unit for hydrogen production. Overall, we find that the prices of these key system components exhibit learning rates in the range of about 9–39%. In particular, we observe that the rate of learning is slowest at 9% for onshore wind turbines based on data for prices and deployment from 1983–2019, and fastest at a rate of 39% for solar PV modules based on data between the years 2011–2019, almost double the traditional 20% learning rate in Swanson’s chart shown in Fig. 1. In interpreting this finding, it is essential to recall that the learning curve focuses on prices rather than the underlying manufacturing cost. The analysis in Reichelstein and Sahoo (2018) suggests that the sharp decline in photovoltaic modules in the years 2008–2013 was only partially attributable to declines in manufacturing costs.^6^ During that time the PV module industry also went through considerable structural changes, with Chinese manufacturers expanding manufacturing capacity at a rapid pace. The consequence of this structural industry change was that the observed sales prices no longer covered the full cost of the goods produced (Reichelstein and Sahoo 2018).

The second part of our analysis applies the constant elasticity learning model to the so-called *Levelized Cost of Energy* (MIT 2007). In the context of power generation, this cost measure is frequently abbreviated as LCOE, with *E* standing for electricity. Analysts rely on this unit cost measure expressed in dollars per kilowatt-hour (kWh) to rank different generation technologies such as fossil fuel versus renewable power plants. While the LCOE is sometimes simplistically conceptualized as *Total Lifetime Cost Divided by Total Lifetime Energy*, the significance of this metric is that, properly defined, it yields a break-even value (Reichelstein and Rohlfing-Bastian 2015). Investors who sell every unit of energy produced during the lifetime of the asset for LCOE dollars per kWh will break even on their investment after incurring capital expenditures, ongoing operating costs, an appropriate return for debt and equity investors, and accounting for applicable corporate income taxes.

We find that the LCOE of solar photovoltaic and onshore wind energy between the years 2010–2019 exhibited learning rates of about 40% both in Germany and in California. For solar power, this rate is comparable to that estimated for the prices of photovoltaic modules. In contrast, the learning rate for the LCOE of wind energy is much higher than the learning rate attributed to the system prices for wind turbines alone. The explanation for the faster decline in the LCOE values is the emergence of significant “denominator effects”, reflecting that technological progress has also increased the capacity utilization rates for a given solar and wind resource. In the context of solar PV, higher capacity utilization reflects improvements in the efficiency of solar cells as well as better equipment such as the use of trackers (Bolinger et al. 2020). For wind power, the improvement in capacity factors has been even more significant, owing to larger rotor blades and improved materials, which enable the turbines to keep (or start) spinning at lower wind speeds (Wiser et al. 2020). Finally, the lifetime energy measure in the denominator of the LCOE expression is increasing in the applicable discount factor, a variable that has also been increasing due to a lower cost of capital for renewable energy (Steffen 2020).

Our analysis also estimates a learning curve for the life-cycle cost of producing hydrogen via electrolysis where electricity splits water into its constituent atoms of hydrogen and oxygen. The corresponding cost measure here is the levelized cost of hydrogen (LCOH) (Glenk and Reichelstein 2021b). We find that for hydrogen the cost improvements over time are again compounded by the interaction between a numerator effect reflecting the declining prices for electrolyzers and a denominator effect corresponding to a lower cost of capital. In addition, an increase in the volatility of electricity prices, which represents a variable cost of producing hydrogen, tends to lower the LCOH. Unlike the capacity factors of wind and solar PV, which are given exogenously, electrolyzers can be idled during periods of high electricity prices. As a consequence, higher volatility of electricity prices, accompanied by constant or decreasing average values, tends to lower the overall life-cycle hydrogen production.

The remainder of the paper is organized as follows. Sect. 2 analyzes the dynamics of system prices for four different clean energy technologies. Sect. 3 goes beyond system prices and integrates additional cost drivers by means of exploring the levelized cost of providing energy via solar PV, onshore wind, or hydrogen electrolysis installations. Sect. 4 concludes with a broader perspective on synergies across different clean energy technologies and the implications for a decarbonized energy economy.

## Dynamics of System Prices

### Solar Photovoltaic Modules

The globally installed capacity of solar PV systems has grown from 4 megawatts (MW) in 1976 to 627 000 MW in 2019 (IEA 2020b). Initially, most solar PV systems were smaller-sized rooftop installations until utility-scale facilities began to comprise for the majority of annual capacity additions. Among the 114 GW of solar PV capacity installed in 2019, for instance, 61% were utility-scale, while commercial and industrial facilities accounted for 24%, and the remaining 15% were residential rooftop systems.

Solar photovoltaic systems comprise an array of PV modules and the so-called balance of system (BOS) components. While the modules consist of individual solar cells that convert solar insolation to electricity, the BOS components comprise the power inverter, other electronic components, wiring and cabling, and installation labor. Increasing standardization of individual components and the growing scale of solar PV facilities have allowed the BOS prices to decline as well, albeit at a smaller rate than the prices of modules (IRENA 2020).

The manufacturing process of crystalline silicon PV modules involves five major sequential steps: (i) purification of metallurgical silicon into polysilicon, (ii) growth of polysilicon ingots, (iii) slicing of ingots into wafers, (iv) lithographic layering of wafers to obtain photovoltaic cells, and (v) assembly of cells to modules (Lux Research 2012). Continued process refinements at each of these steps have reduced the share of defective cells, the amount of polysilicon waste, the number of required manual labor hours. For instance, the reduction of silicon waste in step (iii) resulted from the use of thinner wire saws (Reichelstein and Sahoo 2018). Process automation has significantly lowered the amount of manual labor required and increased the overall factory output.

To measure the learning effects associated with manufacturing PV modules, we adopt Wright’s framework based on global average sales prices per Watt (W) of peak power capacity, thus extending the Swanson chart in Fig. 1 past 2010. Our data source for this calculation is based on BNEF (2019a) and covers the years from 1976–2019. Specific values for yearly module prices and installation capacity are listed in Table 4 of the Appendix.

**Fig. 2:**
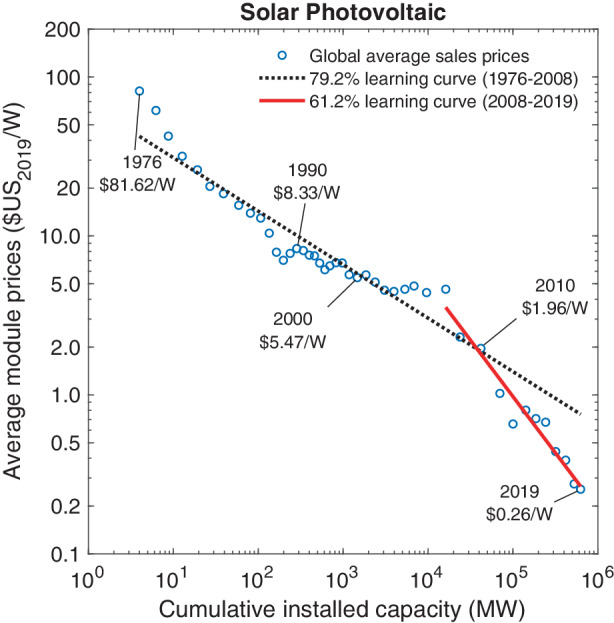
Price dynamics of solar PV modules

Fig. 2 plots the (logarithm of) global average sales prices for solar PV modules against the (logarithm of) global cumulative installation capacity measured in megawatts. Average sales prices for PV modules have fallen from $81.62 W^–1^ in 1976 to $0.26 W^–1^ in 2019. In a minor variant of Swanson’s graph shown in Fig. 1, we estimate that the observed price declines are best described by a 79.2% constant elasticity learning curve for the time window 1976–2008. The corresponding $$R^{2}$$ coefficient for this regression is 0.89.

For a separate regression starting in 2008, however, we observe that module prices dropped much faster than suggested by the historical 80% learning curve. In fact, for the years 2008–2019 we estimate a learning curve of 61.2%, i.e., a learning rate of 38.8% ($R^{2}=0.95$). Earlier studies on the price dynamics of solar PV modules have identified learning curves anywhere in the range of 60–90% (Rubin et al. 2015). As suggested by Fig. 2, the main reason for this variation appears to be the selection of the time window considered. Some of the earlier studies were based only on data from specific geographic regions (Yeh and Rubin 2012). Furthermore, currency fluctuations may have had a significant effect on the resulting parameter estimates. Lilliestam et al. (2020), for instance, find that the choice of currency can lead to differences in the estimated learning rate of up to 16 percentage points.

Many industry analysts have linked the recent steep decline in the prices for photovoltaic modules not only to underlying cost reductions but also to a changing industry landscape characterized by a dramatic increase in the industry’s aggregate manufacturing capacity. To track the dynamics of the cost of manufacturing crystalline silicon photovoltaic modules, Reichelstein and Sahoo (2018) examine the financial statements of about a dozen firms for the years 2008–2013. During that time window, all of the firms in the samples were “pure-play” module manufacturers. Since they had no other significant production activities, their *Cost of Good Sold* and *Finished Goods Inventory* figures reflect only the modules sold and retained by the firm in a particular year.

**Fig. 3:**
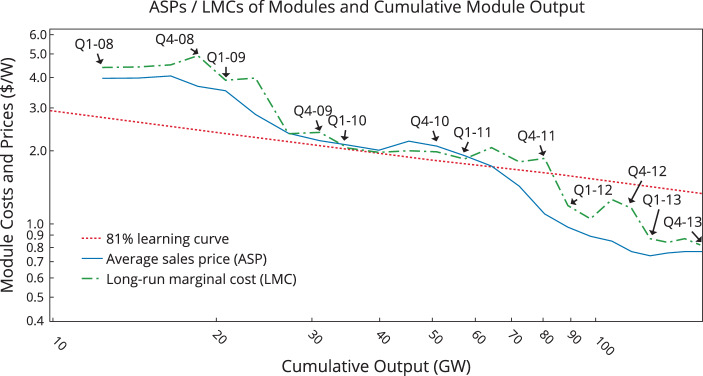
Average sales prices and long-run marginal costs of solar PV modules (Reichelstein and Sahoo 2018)

For the years 2008–2013, Reichelstein and Sahoo (2018) estimate the long-run marginal cost (LMC) of manufacturing solar photovoltaic modules based on firms’ annual reports and industry-level data about module prices and volume. Conceptually, the importance of the LMC is that firms would exactly break even on their investments (achieve a net present value of zero) if modules were to be sold at the current LMC of that year. Furthermore, in a competitive industry in which firms are price takers, the predicted equilibrium price is equal to the LMC in each period. Fig. 3 depicts the actual average module sales prices, the estimated long-run marginal cost, and the “traditional” 80% learning curve. Importantly, actual sales prices were consistently below the estimated LMC, except for a brief period between late 2009 and early 2011. This finding is consistent with the fact that for the years 2008–2013, solar PV manufacturers generally reported negative accounting profits.^7^ The common explanation for prices below the long-run marginal cost during those years is that Chinese manufacturers greatly expanded the production capacity available in the industry during the recessionary phase of the financial crisis.

Overall, the LMC trajectory in Fig. 3 indicates that for the years 2008–2013, both module prices and unit manufacturing costs declined at a faster rate than predicted by the traditional 80% learning curve. Reichelstein and Sahoo (2018) estimate the cost dynamics for two components of the long-run marginal cost of solar PV manufacturing: capacity-related cost for machinery and equipment, and core manufacturing costs for materials, labor, and overhead. Based on quarterly financial statements from a subset of module manufacturers and quarterly data from an industry analyst, the authors infer a 62% constant elasticity learning curve for core manufacturing cost. Capacity-related costs for machinery and equipment were found to have declined at a rate of about 24% per year, again exceeding the traditional long-run 80% learning curve. While it would be insightful to extend this analysis beyond 2013, the inference from publicly observable accounting data has become difficult because most of the leading solar firms have now expanded their product offerings beyond solar PV modules.

Earlier studies on the price dynamics of PV modules have considered potential drivers of learning other than cumulative production volume. Additional potential explanatory variables include raw material prices (e.g., polysilicon and silver), the scale of manufacturing capacity, the number of patents associated with the technology, and expenditures for research and development (Kavlak et al. 2018; Yu et al. 2011; Miketa and Schrattenholzer 2004). The general finding in these studies is that, while other explanatory variables can be statistically significant, these have generally only a minor impact on the estimated coefficient associated with cumulative production volume (Lieberman 1984; Preston and Keachie 1964). In particular, when estimating the 62% learning curve for core manufacturing costs during the years 2008–2013, Reichelstein and Sahoo (2018) control for the scale of the production facilities and substantial price declines in polysilicon. Without these control variables, their analysis yields a coefficient of 59% on cumulative volume over the same time period.

### Onshore Wind Turbines

The pace of installations for onshore wind turbines has accelerated sharply over the past 30 years, growing from 8 MW in 1980 to 650 000 MW in 2019 (Pitteloud 2019). The most recent annual capacity additions have averaged about 50 GW. Onshore rather than offshore wind has thus far accounted for the majority of total annual capacity additions. Of the 61 GW of newly installed wind capacity in 2019, approximately 55 GW were built onshore (Pitteloud 2019; GWEC 2019).

Wind turbines consist of a steel tower, a rotor with blades, and a nacelle containing the drivetrain, a converter and transformers. In contrast to solar PV systems, these components combine both the power generation unit and the BOS components. The steel tower, the nacelle, and the rotor blades are typically constructed individually at the production site of the turbine manufacturer and then transported to and assembled at the location where the wind turbine is to be raised (EERE 2020).

Cost reductions for wind turbines mainly originate from technological improvements that enabled turbine manufacturers to significantly increase wind tower heights and blade lengths. Advanced turbine control systems now make it possible to manage the additional thrust at greater tower heights and to allow for a smooth operation at peak efficiency under conditions of varying wind speeds (Thresher et al. 2008). In general, larger turbines entail additional costs for material and transportation, but also greater peak power generation capacity. The resulting system prices on a per Watt basis have declined.

We calibrate Wright’s functional specification based on the global average system price per Watt of peak capacity for installed onshore wind turbines. In contrast to manufacturing cost of turbines, data on system prices are available over a long time horizon and on a global level. System prices comprise the market prices for the turbines as well as the cost of installation, grid connection, and project development (IRENA 2020). Our analysis relies on data by IRENA (2020) for the turbines, and on the Wind Power Statistic of the World Wind Energy Association for cumulative installed capacity (Pitteloud 2019). Details are provided in Table 4 in the Appendix.

**Fig. 4:**
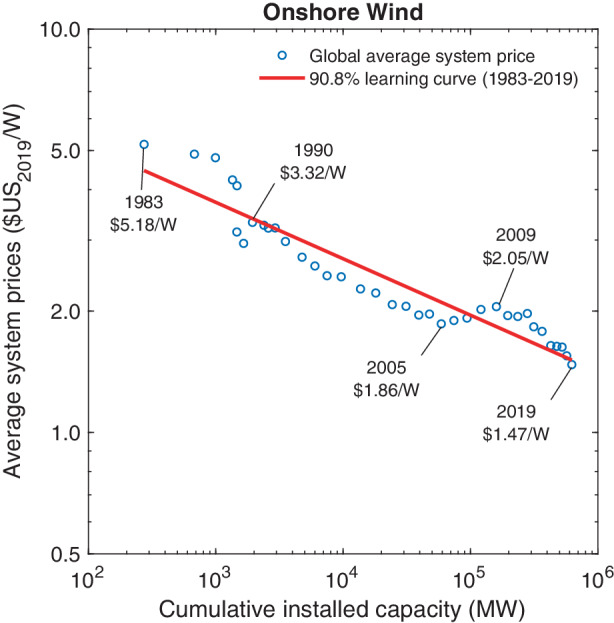
Price dynamics of onshore wind turbines

Fig. 4 depicts the (logarithm of) global average system prices for onshore wind turbines as a function of the (logarithm of) global cumulative installed capacity. Average system prices have fallen from $5.18 W^–1^ in 1983 to $1.47 W^–1^ in 2019. This price trajectory corresponds to a 90.8% learning curve ($$R^{2}=0.89$$), implying that system prices declined by about 9% with every doubling of cumulative installed capacity. We note that the estimated learning rate for the system prices of wind turbines was less than half of that for solar PV modules. This comparison is, however, somewhat misleading since the figures for wind power systems also comprise the BOS components, while our analysis for solar power focused only on modules.

The “spike” in turbine prices during the 2005–2009 time window has been attributed to regulatory shocks pertaining to the extension of subsidies for wind energy in the United States and Europe (Bolinger and Wiser 2012). Specifically, the main subsidy for wind energy in the United States, the federal Production Tax Credit, was widely expected to expire by the end of 2005. When the U.S. Congress agreed to extend this tax credit at relatively short notice, demand for wind turbines and sales prices surged.^8^ With the arrival of the financial crisis in 2008/09, however, demand for wind turbines dropped again and competition among turbine manufacturers intensified, inverting the short-lived upward price trend (Bolinger and Wiser 2012).

It would be desirable to identify the extent to which the decline in average system prices of wind turbines reflects a corresponding decline in the underlying production costs. To base such an analysis on publicly available data, however, is made difficult by recent industry consolidations and the trend among turbine manufacturers to expand their product lines. The number of suppliers in this industry dropped from 63 in 2013 to 33 in 2019 (REN21 2020). Furthermore, major manufacturers for onshore wind turbines have other significant business segments, such as services for operation and maintenance, or the development of offshore wind parks. Their annual financial reports therefore do not provide sufficiently detailed segment information to analyze the cost of onshore wind turbines alone (Vestas 2019; GE 2019; SGRE 2019; Goldwind 2017).^9^ Industry analysts have argued that profit margins for turbine manufacturers have declined substantially in recent years (GWEC 2019; Reuters 2019). This decline has been partly attributed to the shift from feed-in-tariffs to competitive auction mechanisms in countries like China, Germany, and Denmark.

While our calculations point to a 9% constant elasticity learning curve for the market prices of wind turbines, earlier studies have yielded a range between 3% and 30% (Ibenholt 2002; Junginger et al. 2005; Williams et al. 2017). We attribute this variation in part to the fact that some studies have examined country-specific learning curves based on national system prices and/or national capacity deployment (Lindman and Söderholm 2012; Rubin et al. 2015; Williams et al. 2017; Hayashi et al. 2018). Additional variation results from studies that attempt to infer global average system prices based on data from select countries, e.g., the United States, Germany, or Denmark, all of which have recently deployed significant amounts of wind energy (Isoard and Soria 2001; Junginger et al. 2005; Neij 2008). Some earlier studies have looked at global price and capacity data but only for relatively short time windows (Jamasb 2007; Nemet 2009).

### Battery Packs

Energy storage is generally viewed as crucial for a reliable energy supply based on intermittent and volatile power generation sources, such as wind and solar power (Baumgarte et al. 2020). In the transportation sector, inexpensive batteries are key to the electrification of road vehicles, as batteries currently account for about 35% of the sales price of an electric vehicle (BNEF 2019a; Comello et al. 2021). With recent advances in battery technology, deployments of lithium-ion (Li-ion) batteries have grown rapidly in both stationary and mobile applications. In terms of global installed capacity, the energy storage capacity of batteries deployed has grown from 426 megawatt-hours (MWh) in 2010 to 351 000 MWh in 2019 (BNEF 2019b; Schmidt et al. 2019). Electric vehicles have contributed substantially to this growth, with the number of vehicle registrations increasing from 19,000 in 2010 to 7.2 million in 2019 (IEA 2020a).

Li-ion battery systems entail a battery pack that comprises an array of cells protected by a frame. The cells consist of an electrolyte, a separator, and an electrode typically based on graphite (SDI 2021). A battery also requires balance of system components such as the electronic battery management system, electric connections, and a cooling system. Battery packs are the energy component of a battery, reflecting the total amount of energy that can be stored in the battery. The size of this component is measured in Watt-hours (Wh). In contrast, the power component of a battery, which comprises the remaining BOS parts, is measured in Watts and indicates the maximum rate of charge or discharge for the battery. In the context of an electric vehicle, the power component of a battery dermines the vehicle’s ability to accelerate, while, the energy capacity determines the vehicle’s maximum range on a single charge. The ratio of the energy to the power component is generally referred to as the duration of the battery. Duration thus indicates the number of hours for which the battery can charge/discharge at maximum power.^10^

Cost reductions for Li-ion battery packs have resulted from ongoing technological and process improvements at the five main stages of production: (i) manufacturing of the electrode, (ii) cell assembly, (iii) cell finishing, (iv) packing of cells into modules, and (v) aggregation of modules into packs (Heimes et al. 2018). Cost improvements have been attributed to technological advancements in battery cathode chemistry and materials, higher energy density, as well as reduced battery degradation rates. In addition, there have been improvements in the form of vertically integrated production steps that increased manufacturing efficiency. Furthermore, basic economies of scale through so-called gigafactories (Motors 2014) appear to have further contributed to the overall cost and price decline in Li-ion battery packs (Tsiropoulos et al. 2018; Curry 2017).

**Fig. 5:**
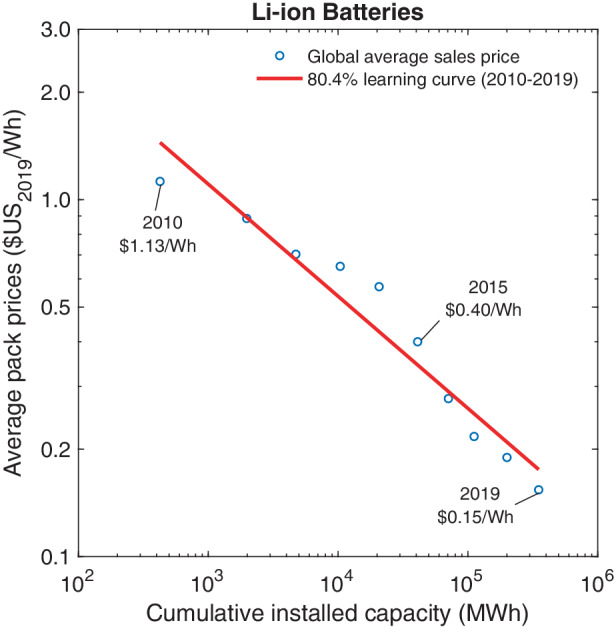
Price dynamics of Li-ion battery packs

Our estimation of the learning curve for Li-ion battery packs relies on data shown in Table 4 in the Appendix. We again rely on market prices for battery packs rather than manufacturing costs due to limited data available for production costs. Pack prices are based on data from BNEF (2020) and Comello and Reichelstein (2019). Global cumulative capacity installation data have been obtained from BNEF (2019b) and Schmidt et al. (2019). Fig. 5 depicts the resulting learning curve, again on a logarithmic scale. Between 2010 and 2019, average sales prices declined by almost 90% from $1.13 Wh^–1^ to $0.15 Wh^–1^. The trajectory of observed market prices yields an 80.4% learning curve ($$R^{2}=0.94$$).

Since our analysis reported in Fig. 5 focuses on Li-ion battery packs designed for large-scale applications in automobiles and grid-level energy storage, we are confined to observations from 2010 onward. Data for battery packs in consumer electronics have been available for a longer time. Such battery packs, however, have a different performance profile in terms of power-to-energy rating, charging and discharging speed, energy density, and longevity. As a consequence, they differ in several aspects, such as component materials, cell designs, cell packing, and hence their overall production costs.

Due to the relative novelty of Li-ion batteries in the power and mobility sector, there have thus far been only a few studies on the applicable learning curves. Based on global average sales prices between the years 2010–2016, Schmidt et al. (2017b), for instance, estimate an 84% learning curve for Li-ion battery packs. Alternatively, Ziegler and Trancik (2021) calculate a learning curve of 76% for cylindrical Li-ion battery cells, which are the most common cell type for grid-level and automobile storage systems.^11^ Furthermore, Kittner et al. (2017) examine how annual production capacity in combination with the number of international patents drive the price decline of Li-ion battery cells of consumer electronics. For the time window 1991–2015, their two-factor estimation model estimates a 83% learning curve associated with the doubling of annual production and a 2% price reduction per 100 patents registered by the Patent Cooperation Treaty.

### Electrolyzers

It has long been observed that hydrogen has considerable potential as a universal energy carrier. The gas can be used for energy storage and the subsequent production of heat and electricity (Staffell et al. 2019). Other important applications include hydrogen as a fuel for transportation and as a feedstock in chemical and processing industries. Widespread adoption of hydrogen in the energy system has so far been held back by the inability to produce the gas without carbon emissions and at low cost.^12^ Recent technological innovations in the form of water electrolysis, whereby (renewable) electricity infused in water splits the water molecule into oxygen and hydrogen (Davis et al. 2018), has renewed the interest in hydrogen.^13^

Leading electrolysis technologies in the market currently include polymer electrolyte membrane (PEM) electrolyzers, alkaline electrolyzers, and solid oxide cell electrolyzers (Staffell et al. 2019). Among those, PEM electrolyzers have exhibited the highest deployment rates in recent years (IEA 2019). One advantage of PEM electrolyzers is their ability to ramp up and down quickly, thus allowing for an almost instantaneous absorption of surplus electricity from the grid during peak hours of renewable power generation. A PEM electrolyzer consists of multiple electrolysis stacks, which are surrounded by a balance of system comprising thermal and fluid management, power electronics, and hydrogen treatment (Schmidt et al. 2017a). Each stack is made up of several cells in which two electrodes, separated by a membrane, split the water molecule into oxygen and hydrogen.

Since the production of PEM electrolyzers has thus far originated in customized contract manufacturing, the process has traditionally been relatively labor intensive (Schmidt et al. 2017a). Some cost reductions have resulted from early efforts of standardization and automation of production processes as well as growing sizes of production plants. Technological advancements have further reduced overall input material cost and production waste. Examples of these advancements include improved electrode design, membranes and catalyst coating, and the substitution of expensive input materials with cheaper yet more efficient materials.

**Fig. 6:**
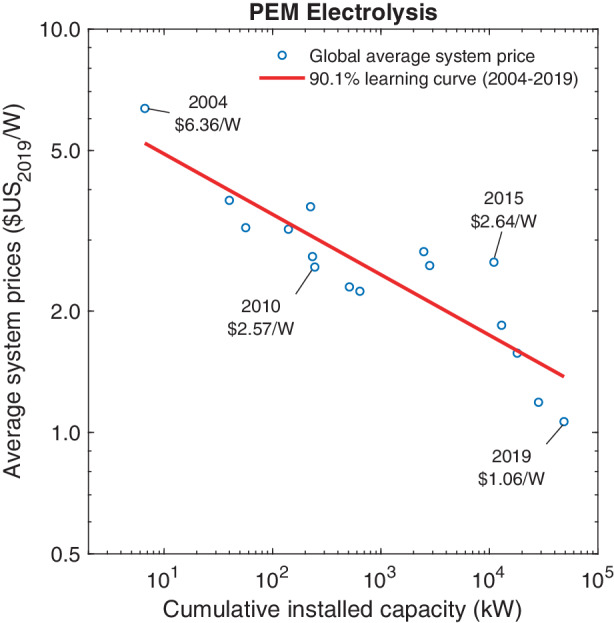
Price dynamics of PEM electrolyzers

In estimating the learning curve for PEM electrolyzers, we rely on global average system prices that were hand-collected from journal articles, technical reports, and interviews with manufacturers (Glenk and Reichelstein 2020). Detailed information regarding the underlying manufacturing costs has remained scarce. Data on cumulative capacity installations is based on a comprehensive review of Power-to-Gas electrolyzer facilities deployed around the world (IEA 2019). Fig. 6 shows the corresponding Wright (1936) learning curve. Prices have fallen from $6.36 W^–1^ in 2004 to $1.06 W^–1^ in 2019. We estimate a 9.9% reduction in prices with every doubling of volume. The $$R^{2}$$ in this regression is 0.79. Direct comparison of the graphs in Figs. 2–6 shows that the prices of PEM electrolyzers exhibited greater variation from the projected trajectory, which we attribute to PEM electrolyzers becoming commercially viable only within the last 15 years.

## Dynamics of the Levelized Cost of Energy

The economics of power generation facilities varies not only with the generation technology employed but also with the size and location of the facility. To capture and compare the unit economics of competing facilities, the energy literature has focused on life-cycle cost measures.^14^ In the context of electricity, this unit cost is frequently referred to as the levelized cost of electricity (LCOE). Expressed in dollars per kWh, the LCOE identifies the unit revenue that an investor in an energy facility would have to obtain on average over the useful life of the asset to break even in terms of discounted cash flows (MIT 2007). As such, the LCOE allows for a cost comparison of alternative power generation technologies that differ in terms of their cost structure and operational characteristics across the lifetime of the asset, e.g., natural gas turbines vs. solar PV installations.^15^

Following the concept development in Reichelstein and Rohlfing-Bastian (2015) and Comello et al. (2020), the LCOE can be expressed as the sum of three components:

2 $$\text{LCOE}=w+f+c\cdot\Delta.$$

Here, $w$ and $f$ denote the (levelized) variable and fixed operating costs per kWh, respectively. These costs are either zero or minor for solar photovoltaic and wind power. The unit cost of capacity, $c$, is obtained by “levelizing” the initial systems price:

3 $$c=\frac{\text{SP}}{8760\cdot\text{CF}\cdot\sum_{i=1}^{T}x_{i}\cdot(\frac{1}{1+r})^{i}}.$$

For instance, in the context of wind energy, the numerator, SP, in the definition of the unit cost of capacity refers to the cost of acquiring, installing, and connecting wind turbines (in $ kW^–1^). Thus, our calculations below will refer back to the system prices shown in Fig. 4 above. The levelization factor in the denominator is the product of two components. The product $$8760\cdot\text{CF}$$ yields the effective number of hours per year that the facility is generating electricity at its nameplate capacity.^16^ The scalar $$\sum x_{i}\cdot(\frac{1}{1+r})^{i}$$ reflects the number of years the generation facility will be in operation ($$T$$). This number is “discounted” at the applicable cost of capital, $$r$$, and the degradation factor, $$x_{i}$$, which reflects that the asset may diminish in productive generation capacity over time.^17^

Finally, the tax factor $\Delta$ quantifies the financial impact of corporate income taxes, the allowable depreciation schedule for tax purposes, and any applicable investment tax credits. For instance, the United States tax code currently grants an investment tax credit for solar PV installations and for energy storage systems, in particular batteries, that are installed in connection with solar systems (U.S. Department of Energy 2016). This credit is calculated as a percentage of the system price that is deducted from the investor’s income tax liability. Tax shields for debt financing are included in the calculation of the cost of capital as this number will be calculated as the weighted average cost of capital (Ross et al. 2008).

### Levelized Cost of Electricity

We first examine the dynamics of the LCOE for utility-scale solar PV and onshore wind power installations in the context of California and Germany.^18^ Both jurisdictions have deployed considerable amounts of renewable energy in recent years, with utility-scale solar facilities being added since around 2010. By international comparison, California and Germany have moderate wind resources. In contrast to Germany, California enjoys a high degree of insolation.

Table 1 lists parameter values for the main input variables for the years 2010 and 2019. System prices for solar PV include the market prices for modules, BOS components, and the cost of installation. Similarly, the acquisition cost of turbines and their installation comprise the system prices for wind energy. Our calculations rely on data collected from multiple sources including industry databases, technical reports, and journal articles. A comprehensive list of all input and output variables is provided in the Appendix Tables 5–8.

**Table 1:** Cost parameters for renewable energy sources

	California		Germany	
In 2019 $US	2010	2019	2010	2019
Solar PV				
System price ($ kW^–1^)	5396	1343	3705	899
Fixed operating cost ($ kW^–1^)	14.03	8.81	35.51	7.34
Capacity factor (%)	21.04	28.69	7.44	10.80
Cost of capital (%)	6.04	4.50	4.60	2.00
Useful lifetime (years)	30	30	30	30
Federal tax rate (%)	35.00	21.00	30.00	30.00
Onshore Wind				
System price ($ kW^–1^)	2927	1678	2271	1762
Fixed operating cost ($ kW^–1^)	28.75	21.94	73.00	48.88
Capacity factor (%)	27.84	34.70	24.00	31.10
Cost of capital (%)	6.04	4.50	4.60	2.00
Useful lifetime (years)	30	30	30	30
Federal tax rate (%)	35.00	21.00	30.00	30.00

While the overall system prices for both photovoltaic systems and wind turbines have declined dramatically between 2010 and 2019, different components declined in price at different rates. For solar photovoltaic systems, for instance, the decline in PV module prices was diluted by BOS costs falling at a slower rate.^19^ Specifically, the relative share of modules within the total system price declined from 41.7% in 2010 to 25.6% in 2019 (IRENA 2020; BNEF 2019b). One cause of this shift is the increasing deployment of axis trackers (Bolinger et al. 2020). By enabling PV modules to track the sun across the hours of the day, trackers also yield an increase in the capacity factor, CF, in the denominator of Eq. (3). For wind turbines, capacity factors also increased mainly as a result of the growing turbine towers and rotor blades, which allows the turbines to convert wind at higher altitudes and lower wind speeds.

Table 1 also indicates that the cost of capital for renewable energy investments decreased substantially over the past decade (Steffen 2020). This reduction can be attributed not only to the recent decline in interest rates but also to the fact that over time debt and equity investors appear to require a lower risk premium for renewable energy investments (Egli et al. 2018). A lower cost of capital in the denominator of the unit capacity cost $c$ in Eq. (3) again contributes to a lower LCOE. Finally, the U.S. federal government implemented two changes to the federal tax code that came into effect in 2018: the corporate income tax rate was lowered from 35.0% to 21.0%, and upfront capacity expenditures for new energy facilities can be depreciated fully in the year of investment (U.S. IRS 2019).

**Fig. 7:**
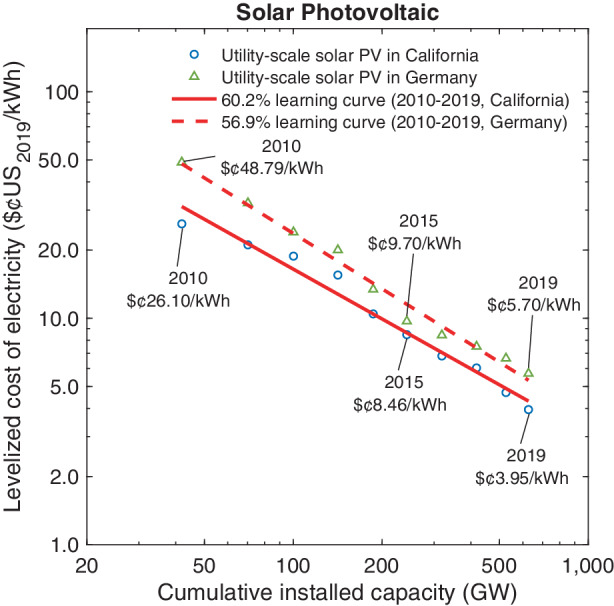
LCOE Dynamics: Solar Power

The trajectory of the logarithmic LCOE values for utility-scale solar PV in California and Germany are shown in Fig. 7. As before, we plot these values as a function of cumulative capacity installations, even though there has been little empirical support for the notion that all components of the LCOE are described reasonably well according to the classic learning curve model. In fact, for certain components, such as the capacity factor, CF, it appears unlikely that this variable changes with a constant elasticity function for increases in cumulative volume. Nonetheless, Fig. 7 indicates that the aggregate LCOE values for the decade spanning the years 2010–2020 conform quite closely to a 60.2% learning curve in California ($R^{2}=0.97$) and a 56.9% learning curve in Germany ($R^{2}=0.99$). We regard it as a coincidence that the predicted learning for the LCOE of solar PV power is almost identical to that for solar modules alone, as shown for the decade 2008–2019 in Fig. 2. The differences in the LCOE values between the two jurisdictions shown in Fig. 7 mainly reflect the better solar insolation factors and correspondingly higher capacity factors in California.

While the parameter value of 2.0% for the cost of capital in Germany is based on the literature sources we used throughout our calculations, this figure may intuitively seem too low for investments in clean energy technologies, despite the possibility of highly leveraged investments and the presence of impact equity investors. We note that if this cost of capital were to be set at 3.0% in 2019, the LCOE value for solar PV would increase from $¢5.70 kWh^–1^ to $¢6.50 kWh^–1^.^20^ We also note that the capacity factors we impute are based on national solar insolation averages. In both Germany and the U.S., southern locations yield substantially better than average capacity factors. Finally, we note that the values in Fig. 7 exclude public policy support that is available for solar installations in California through the federal investment tax credit and in the form of a feed-in premium for Germany. Accounting for the investment tax credit, our calculations yield LCOE values of $¢2.66 kWh^–1^ in 2019 for California.

**Fig. 8:**
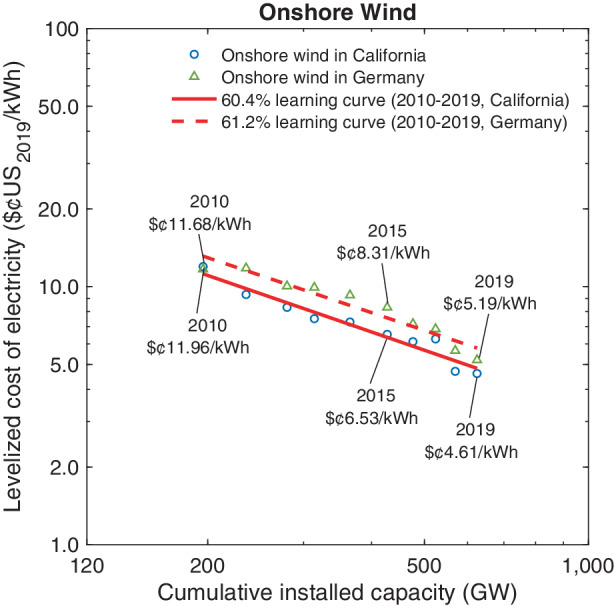
LCOE Dynamics: Wind Power

Fig. 8 plots the equivalent of Fig. 7 for onshore wind capacity. In California, the LCOE values declined from $¢11.96 kWh^–1^ in 2010 to $¢4.61 kWh^–1^ in 2019, and from $¢11.68 kWh^–1^ to $¢5.19 kWh^–1^ in Germany over the same time. As we did for solar photovoltaic systems, public subsidies for wind energy are excluded in both jurisdictions.^21^ The relatively small difference between LCOE values for the two jurisdictions result primarily from higher fixed operating costs in Germany, partially compensated by a lower cost of capital.

Our finding of a 60.4% learning curve in California ($R^{2}=0.94$) and a 61.2% learning curve in Germany ($R^{2}=0.92$) for onshore wind is surprising in light of our earlier finding in Fig. 4 that between 1983–2019 wind turbines experienced “only” a 90.8% learning curve in terms of system prices. One explanation for the much faster drop in LCOE values is that the regression analysis underlying Fig. 8 is based on a much shorter time horizon.^22^ A second explanation for the faster drop in the LCOE is through the denominator in Eq. (3). Here, capacity factors have increased sharply due to better building materials resulting in higher yields of converting wind energy into electric power.^23^ Finally, our data sources indicate that the weighted cost of capital for wind projects declined significantly and thereby increased the term $\sum_{i=1}^{T}x_{i}\cdot(\frac{1}{1+r})^{i}$ in Eq. (3).

Our estimates of the LCOE dynamics shown in Figs. 7 and 8 is generally consistent with LCOE values reported by industry analysts and academic studies; see, for instance, Bolinger et al. (2020), Wiser et al. (2020), Kost and Schlegl (2010), and Kost et al. (2018). Importantly, even excluding policy support for renewables, onshore wind and solar PVs have now attained lower LCOE values than traditional power generation technologies powered by coal or natural gas. To illustrate, in 2019 the LCOE of a potential new brown coal power plant, the cheapest fossil power source in Germany, ranged between $¢5.5 kWh^–1^ and $¢9.6 kWh^–1^ depending on the respective location (Kost et al. 2018). In California, the LCOE of natural gas combined-cycle plants lies in the range of $¢5.8–8.0 per kWh in 2019 depending on the capacity utilization rate (Neff 2019; Comello et al. 2020; Glenk and Reichelstein 2021a).

### Levelized Cost of Hydrogen

In direct analogy to the LCOE concept, we finally examine the dynamics of the life-cycle cost of hydrogen (LCOH) production, when the hydrogen is produced through water electrolysis. The LCOH is defined as the critical dollar value per kg of hydrogen that allows an investor to break even in terms of discounted after-tax cash flows over the useful life of the electrolyzer (Glenk and Reichelstein 2021b). Aside from the initial equipment cost, applicable fixed operating costs include maintenance and spare part replacements. In contrast to renewable energy generation, electrolysis requires significant variable processing costs due to the consumption of electricity.

In further contrast to wind and solar photovoltaic power, the capacity factor of an electrolyzer is determined endogenously rather than being given exogenously by the availability of the respective natural resource. For the electrolyzer to operate efficiently, the conversion value of hydrogen, defined as the amount of hydrogen obtained per kWh less any ancillary expenses for iodized water, must at any given hour exceed the price of electricity at that time. The contribution margin of hydrogen thus varies over time and hinges on the price of electricity as well as the time-invariant conversion rate that determines the number of kilograms of hydrogen obtained from one kWh of electricity (Glenk and Reichelstein 2020).

We examine the dynamics of the LCOH in the context of PEM electrolyzers deployed in Germany, where most of the demonstration projects have been built to date (IEA 2019). Table 2 shows average values of the main cost parameters for PEM electrolysis facilities in the years 2010 and 2019 (further details are provided in the Appendix in Table 9). The system prices correspond to those reported in Fig. 6. Fixed operating costs are estimated as a percentage of the system cost.

**Table 2:** Cost parameters for PEM Electrolysis

	Germany	
In 2019 $US	2010	2019
System price ($ kW^–1^)	2571	1064
Fixed operating cost ($ kW^–1^)	77.13	31.91
Hydrogen conversion rate (kg kWh^–1^)	0.0166	0.0192
Average electricity buying price ($¢ kWh^–1^)	6.20	4.42
Cost of capital (%)	4.60	2.00
Useful lifetime (years)	25	25

The conversion rates reported in Table 2 originate from interviews with industry experts and are assumed to have increased linearly between the years 2010–2019 (IEA 2019; Glenk and Reichelstein 2019). The cost of capital is taken to be the same as for wind energy in Germany, because electrolyzers are frequently co-located with wind power plants. The electrolysis is assumed to rely on electricity from the wholesale power market. Electricity purchases for water electrolysis are exempt in Germany from most taxes and fees paid by other industrial customers (EEG 2020). Our calculations rely on hourly electricity prices in the day-ahead wholesale market.

**Fig. 9:**
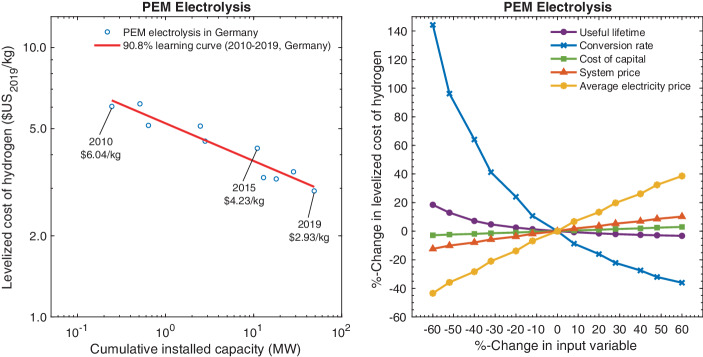
LCOH Dynamics and Sensitivity

Invoking again Wright’s concept of learning-by-doing, Fig. 9 shows the trajectory of the (logarithm of) LCOH values for PEM electrolysis in Germany as a function of the (logarithm of) global cumulative installed capacity. We find that the LCOH values have fallen from about $6.04 kg^–1^ in 2010 to $2.93 kg^–1^ in 2019. This decline yields an estimate of a 90.8% learning curve, similar to that of the system prices for PEM electrolyzers shown in Fig. 6. This alignment of learning curves may seem counter-intuitive in light of the fact that the variable cost of hydrogen production corresponding to electricity, i.e., $$w$$ in Eq. (2), is significant, and our argument that the LCOE of renewable energy experienced learning curves of around 60% over the past decade. The explanation here is that our calculations in Fig. 9 are based on wholesale market prices in Germany, yet these declined at a much slower rate than the LCOE of wind and solar PV.

Since the preceding calculations rely on several crucial parameter estimates, it is instructive to examine the sensitivity of the findings to changes in the key input variables. Fig. 9 presents a sensitivity analysis in the form of a “spider diagram” based on cost and price parameters for the year 2019. The LCOH for PEM electrolysis is most sensitive to the conversion rate as this rate determines not only the amount of hydrogen obtained from 1 kWh of electricity but, by implication, also the efficient capacity utilization rate, i.e., the capacity factor. As one might expect, the average electricity price is also a central driver of the LCOH, reflecting that the price of electricity drives both the variable cost of hydrogen production and the resulting capacity factor. The relative insensitivity of the LCOH to changes in the cost of capital reflects that variable costs, rather than upfront investment, account for the majority of the life-cycle cost.

While our analysis focused on electrolysis facilities operating as stand-alone units, many electrolyzers currently deployed are co-located with a renewable energy source (IEA 2019).^24^ Such vertical integration enables the transfer of renewable power to the electrolyzer and entails operational synergies whenever the price for buying electricity from the grid exceeds the selling price faced by the renewable source. Glenk and Reichelstein (2020) demonstrate that such synergistic benefits can cause a vertically integrated electrolyzer to break even at a substantially lower price for hydrogen than a stand-alone electrolyzer feeding on grid electricity.

## Concluding Remarks

It is widely acknowledged that the economics of carbon-free energy generation has improved substantially in recent years as these relatively new technologies have been deployed at an accelerating pace. Solar photovoltaic modules provide a prime example of a price trajectory for which the 80% constant elasticity learning curve has proven highly descriptive over the time period 1976–2008. While observers frequently voiced concern about the possibility of extending this rate of price reductions indefinitely, the past decade has seen price declines for solar PV modules at rates that are substantially faster than the traditional 20% rate.^25^

Our analysis in this paper has shown that the learning curves of the levelized cost of energy are, in some instances, substantially faster than those observed for the system prices of the underlying clean energy technology, i.e., wind turbines or solar PV systems. This finding primarily reflects a “denominator effect” in the calculation of the LCOE. Technological progress has not only lowered the cost of producing the power generation system, represented in the numerator of the LCOE, but also increased the capacity factor, represented in the denominator, due to better conversion rates for the available wind or solar resources.

**Fig. 10:**
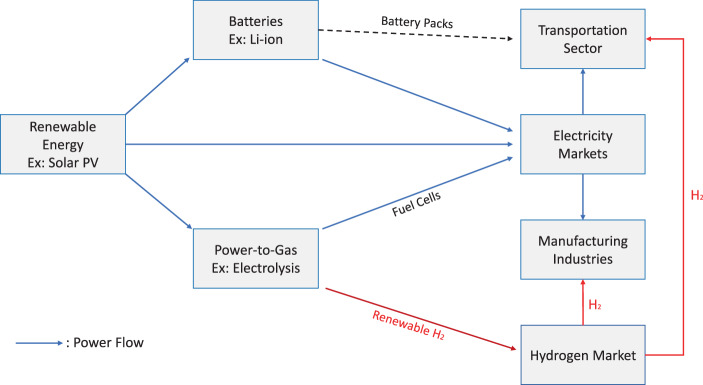
Interdependencies of Learning Effects

From the perspective of the overall transition to a carbon-free energy system, we note that there are significant economic synergies between the technologies examined in this paper. For instance, the anticipated future learning effects for both renewable energy and lithium ion battery packs have a compounding effect in terms of the levelized cost per mile driven for battery electric vehicles (Comello et al. 2021). Similarly, as illustrated in Fig. 10, the cost of green hydrogen obtained through electrolysis will be pushed down not only by lower electrolyzer prices but also by cheaper renewable energy feeding the electrolysis process. Moving further afield, inexpensive green hydrogen will make this energy carrier a potentially attractive alternative to coal as a heating agent in industries like cement and steel. Finally, low cost green hydrogen will make drivetrains powered by fuel cells more competitive with internal combustion engines and pure battery-electric vehicles.

Our learning curve framework lends itself to evaluating the plausibility of the cost reduction goals set recently by the Biden Administration in connection with solar photovoltaic energy and hydrogen obtained through electrolysis. As mentioned in the Introduction, Energy Secretary Jennifer Granholm told Congress in April of 2021 that the administration had adopted a goal of cutting the price of solar in half by 2030 and lowering the cost of clean, renewable hydrogen by 80 percent before 2030. The learning curves for the solar LCOE and the LCOH derived in this paper provide an indication of the rate at which these technologies will have to be deployed in order to meet the stated goals.

Extrapolating the constant elasticity learning curve for the LCOE of solar PV systems in California, our estimates suggest that in order to reach the stated goal of cutting the cost of solar in half by 2030, cumulative global installation volume would have to double on average every 7.3 years. To see this, we note that with a 60.4% learning curve Fig. 7, cumulative global volume of solar PV installations would have to double in 7.3 years starting in 2020, and thereafter maintain that growth rate. This follows because $\frac{2030-2020}{7.3}=1.37$ and $(0.602)^{1.37}=0.5$. Thus, global solar installations would have to double from the current 600 GW to around 1200 GW within the next 7.3 years and, on average, maintain that rate through 2030. During the past decade, by comparison, the average time to doubling for solar PV was 3.9 years. Thus, the goal of cutting the cost of solar energy in half by 2030 could be achieved with a rate of deployment growth that is slower in the coming decade than it was in the past decade.

The 80% cost-cutting goal for hydrogen appears particularly ambitious. Referring to our learning curve estimate of 90.8% for electrolysis Fig. 9, cumulative global volume of electrolyzers would, on average, have to double every 0.6 years, because $\frac{2030-2020}{0.6}=16.68$ and $(0.908)^{16.68}=0.2$. In calibrating this required growth rate, it is useful to recall that between 2010 and 2019 the cumulative PEM electrolyzer capacity grew from 0.25 to 49 MW Fig. 9. That, in turn, corresponded to an average doubling time of 0.4 years for that time window. Thus, a slowing growth rate for electrolyzer deployments would still be consistent with the articulated goals, provided the dynamics of the learning curve observed in the past persists in the future.

The immediate follow-on question to these projections concerns the policy incentives that will have to be in place in order for the above deployment growth rates to be attained. As noted above, solar PV installations have taken off in response to investment tax credits, renewable energy portfolio standards and feed-in-tariffs. In contrast, for hydrogen no comparable support mechanism has been in place thus far in the U.S. or in Europe.

## Supplementary Information




## Footnotes

1 Recent estimates by the Intergovernmental Panel on Climate Change (IPCC) leave the world with a carbon budget in the range of 400–800 billion metric tons of CO_2_ in order to stay well the upper bound 2 °C warming goal, with at least a “reasonable” two-thirds chance. For reference, global anthropogenic emissions amounted to approximately 42 billion metric tons of CO_2_ in 2019 (Rogelj et al. 2018).

2 Projections for potential CO_2_ reduction pathways typically rely on learning curves for estimates of technological progress for clean energy technologies; see, for instance, Nordhaus (2019); Luderer et al. (2018).

3 Wright (1936) documented a corresponding constant elasticity relationship between the cumulative number of aircraft frames produced and the number of labor hours required for the next airframe.

4 Fig. 1 shows a temporary bump in solar PV module prices in the years leading up to 2010. This bump was widely attributed to a shortage of the essential raw material polysilicon. By the end of 2010, average sales prices had virtually caught up with the values predicted by the 80% learning curve.

5 This concept refers to the number of transistors on a chip doubling every two years, leading to the recursive relation $T(n+2)=2\cdot T(n)$, where $T(\cdot)$ represents the number of transistors and $n$ the number of years.

6 Reichelstein and Sahoo (2018) estimate that the cost of manufacturing modules declined at a rate of 38%.

7 The LMC includes a charge for the opportunity cost of capital. Therefore, negative accounting profits imply, at least in theory, that prices were below the LMC (Reichelstein and Sahoo 2018).

8 Efiong and Crispin (2007) report a tripling of one manufacturer’s margins on earnings before interest and taxes in the period from 2005–2007.

9 Among the more than 30 firms that are active in this industry, the four leading players (Vestas, Siemens Gamesa, Goldwind, GE Renewables) had 55% of total sales in 2019 (REN21 2020). Furthermore, the leading players are also active in the offshore wind market and in other renewable energy segments.

10 Stationary battery systems, like the Tesla Powerwall, frequently have a duration of four hours.

11 Adjusting for the substantial improvements in volumetric and gravimetric energy density of Li-ion cells, Ziegler and Trancik (2021) even identify a learning curve of 69% for cylindrical Li-ion cells.

12 Industrial hydrogen production currently accounts for over 2% of annual global CO_2_ emissions (IEA 2019).

13 Such electrolytic hydrogen production is of obvious interest from the perspective of buffering the growing volatility in electricity markets resulting from the large-scale deployment of wind and solar energy installations (Olauson et al. 2016; Wozabal et al. 2016).

14 See, for instance, Lazard (2016, 2018, 2020) or NREL (2009).

15 As pointed out by Joskow (2011), comparing average electricity prices to the LCOE of renewable energy is generally misleading, because such comparisons ignore the inherent intermittency of wind and solar power. As a consequence, the revenues obtained by renewables depends will depend on the market prices that prevail at different hours of the day and during different seasons.

16 For solar PV and wind energy, the scalar capacity factor CF is exogenously given by the availability of solar insolation or wind speed in a particular location, assuming the facility will always generate electricity at the maximum rate available.

17 For instance, the efficiency of solar PV modules was observed to decay at a constant annual rate of about 0.5% (Jordan and Kurtz 2013).

18 Clean energy technologies may exhibit external cost such as noise, the reduction of wildlife, or the mining of rare earths (Kaldellis et al. 2016; Leung and Yang 2012; Wu et al. 2020). These external costs may impede the social acceptance of clean energy technologies (Enevoldsen and Sovacool 2016). Since these costs are generally borne by the investor, they are not included in our LCOE calculations.

19 Louwen and van Sark (2020), for instance, find that the reduction in the cost of balance of system components for utility-scale solar PV plants in Germany is described by an 88% learning curve for the years 2006–2017.

20 Online Appendix provides all input parameters as well as the corresponding graphs for the cost of capital amounting to 3.0% in 2019.

21 Some studies include the production tax credit that is available for wind energy facilities in the U.S. in the calculation of the LCOE. Accounting for this credit, our calculations yield LCOE values in California of $¢9.92 kWh^–1^ in 2010 and to $¢3.91 kWh^–1^ in 2019.

22 A regression of global average system prices of wind turbines over the years 2010–2019 yields a learning curve of 83.9% ($R^{2}=0.92$).

23 Schauf and Schwenen (2021) estimate a multifactor experience curve and also find significant learning in capacity factors, which they attribute to learning by searching.

24 Wind power naturally complements electrolyzers as it produces most of its output during the night when demand from the grid and electricity prices tend to be low (Reichelstein and Sahoo 2015).

25 This observation brings to mind Mark Twain’s well-known adage “…reports of my death are greatly exaggerated”.
