# Supplementary material for: Cost Dynamics of Clean Energy Technologies
Source: Schmalenbach Z Betriebswirtsch Forsch. 2021 Sep 7;73(2):179–206. doi: 10.1007/s41471-021-00114-8 (PMC8422065; doi:10.1007/s41471-021-00114-8)

Online Appendix

Symbols and Acronyms

Cost and Price Parameters

Table 4 provides a comprehensive list of input parameters used for estimating the learning curves of system price components of all four clean energy technologies. The corresponding source for each data vector is provided in the last row of the table.

Tables 5–9 provide a comprehensive list of annual input parameters and corresponding sources for our calculations of the LCOE of solar and wind energy in California and Germany. For solar PV and wind power facilities in California, system prices and fixed operating cost reflect average values of respective plants installed across the entire United States in the particular year, adjusted for the price level in California using averaged city cost indexes by RSMeans (2020). System prices for solar PV and wind turbines are taken from Bolinger et al. (2020) and Wiser et al. (2020), respectively. Fixed operating cost are calculated for either energy

Table 3 List of symbols and acronyms

b	Learning parameter
BOS	Balance of system
c	Unit cost of capacity ($\$\,kWh^{-1}$)
CO_2	Carbon dioxide
°C	Degree Celcius
Δ	Tax factor (–)
f	Levelized fixed operating cost ($\$\,kWh^{-1}$)
GW	Gigawatts
IPCC	Intergovernmental Panel on Climate Change
LCOE	Levelized cost of electricity ($\$\,kWh^{-1}$)
LCOH	Levelized cost of hydrogen ($\$\,kg^{-1}$)
kWh	Kilowatt hour
MW	Megawatts
MWh	Megawatt hours
n	Number of years
P	Sales price per unit of equipment
PEM	Polymer electrolyte membrane
PV	Photovoltaic
Q	Quantity of cumulative industry output
$T(\cdot)$	Number of transistors
w	Levelized variable operating cost ($\$\,kWh^{-1}$)
W	Watt
WACC	Weighted average cost of capital
Wh	Watt hours

source from data by ABB (2020). Annual average capacity factors of solar PV and wind energy facilities in California are calculated based on reported hourly capacity factors of individual plants in a particular year as provided by ABB (2020).

For solar PV and wind power plants in Germany, system prices and fixed operating cost reflect average values of respective facilities in Germany in the particular year. System prices for solar PV and wind turbines are collected from IRENA (2020) as

Table 4 Global system prices and cumulative installed capacity

	Solar PV		Onshore Wind		Li-ion Batteries		PEM Electrolysis	
Year	Module Price	Capacity	System Price	Capacity	Pack Price	Capacity	System Price	Capa-city
	($ kW^{-1})	(MW)	($ kW^{-1})	(MW)	($ kWh^{-1})	(MWh)	($ kW^{-1})	(MW)
1976	81 616	4	–	–	–	–	–	–
1977	61 568	6	–	–	–	–	–	–
1978	42 477	9	–	–	–	–	–	–
1979	31 729	13	–	–	–	–	–	–
1980	26 062	19	–	–	–	–	–	–
1981	20 502	27	–	–	–	–	–	–
1982	18 433	39	–	–	–	–	–	–
1983	15 593	59	5179	274	–	–	–	–
1984	13 906	81	4898	680	–	–	–	–
1985	12 951	107	4799	995	–	–	–	–
1986	10 407	135	4229	1354	–	–	–	–
1987	7901	164	4090	1469	–	–	–	–
1988	7028	198	3141	1465	–	–	–	–
1989	7766	238	2943	1655	–	–	–	–
1990	8331	285	3318	1943	–	–	–	–
1991	8093	340	3264	2392	–	–	–	–
1992	7570	400	3211	2587	–	–	–	–
1993	7499	460	3214	2930	–	–	–	–
1994	6750	530	2975	3527	–	–	–	–
1995	6131	610	2720	4763	–	–	–	–
1996	6488	699	2587	6007	–	–	–	–
1997	6785	825	2447	7482	–	–	–	–
1998	6761	978	2432	9667	–	–	–	–
1999	5704	1179	2269	13 700	–	–	–	–
2000	5465	1469	2217	18 039	–	–	–	–
2001	5690	1860	2075	24 322	–	–	–	–
2002	5126	2385	2055	31 181	–	–	–	–
2003	4556	3075	1955	39 295	–	–	–	–
2004	4477	3971	1966	47 678	–	–	6360	0.007
2005	4620	5344	1859	59 009	–	–	3762	0.040
2006	4842	6845	1895	74 109	–	–	3219	0.057
2007	4402	9618	1921	93 916	–	–	3191	0.140
2008	4622	16166	2018	120 888	–	–	3628	0.224
2009	2313	23 833	2051	159 744	–	–	2730	0.233

Table 4 (Continued)

2010	1961	41 925	1949	196 104	1125	426	2571	0.245
2011	1022	70 240	1939	235 184	886	1982	2295	0.511
2012	656	100 201	1972	279 670	703	4734	2239	0.639
2013	802	141 661	1828	313 882	651	10 396	2807	2.480
2014	709	186 661	1781	364 978	571	20 714	2593	2.812
2015	673	242 661	1642	427 244	400	41 036	2643	11.000
2016	440	318 972	1635	476 300	277	70 805	1844	12.951
2017	389	417 972	1628	524 137	217	111 682	1572	18.045
2018	275	525 972	1549	569 596	190	200 000	1188	28.369
2019	255	626 920	1473	624 626	154	351 000	1064	48.713
Sources	[1]	[1]	[2]	[2, 3]	[4, 5]	[1, 6]	[7]	[8]

[1] BNEF (2019b), [2] IRENA (2020), [3] Pitteloud (2019), [4] BNEF (2020), [5] Comello and Reichelstein (2019), [6] Schmidt et al. (2019), [7] Glenk and Reichelstein (2019, 2020), [8] IEA (2019), $-values are in 2019 $US

Table 5 Levelized cost dynamics for solar PV in California

In 2019 $US	Source	2010	2011	2012	2013	2014	2015	2016	2017	2018	2019
Input Parameters											
Useful lifetime (years)	[1]	30	30	30	30	30	30	30	30	30	30
System price ($ kW^{-1})	[9]	5396	4485	4088	3504	2967	2593	2161	1986	1565	1343
Fixed operating cost ($ kW^{-1})	[2]	14.03	12.21	12.39	12.90	11.28	9.15	6.92	6.79	8.27	8.81
Variable operating cost ($¢ kWh^{-1})		0.00	0.00	0.00	0.00	0.00	0.00	0.00	0.00	0.00	0.00
Capacity utilization rate (%)	[2]	21.04	21.04	20.83	21.78	26.75	27.67	27.67	29.23	29.59	28.69
Cost of capital (%)	[3,10]	6.04	5.78	5.47	5.46	5.25	4.92	4.68	4.80	5.15	4.50
Degradation factor (%)	[1, 4, 5]	99.50	99.50	99.50	99.50	99.50	99.50	99.50	99.50	99.50	99.50
Investment tax credit (%)		0.00	0.00	0.00	0.00	0.00	0.00	0.00	0.00	0.00	0.00
ITC capitalization (%)		50.00	50.00	50.00	50.00	50.00	50.00	50.00	50.00	50.00	50.00
Production tax credit ($¢ kWh^{-1})		0.00	0.00	0.00	0.00	0.00	0.00	0.00	0.00	0.00	0.00
State tax rate (%)	[6]	8.84	8.84	8.84	8.84	8.84	8.84	8.84	8.84	8.84	8.84
Federal tax rate (%)	[7]	35.00	35.00	35.00	35.00	35.00	35.00	35.00	35.00	21.00	21.00

Table 5 (Continued)

State tax depreciation method (–)[a]	[8]	3	3	3	3	3	3	3	3	3	3
Federal tax depreciation method (–)[a]	[8]	2	2	2	2	2	2	2	2	5	5
Levelized Cost											
Cost of capacity, c ($¢ kWh^{-1})		22.47	18.18	16.20	13.26	8.94	7.28	5.91	5.21	4.22	3.47
Tax factor, Δ (–)		1.13	1.12	1.12	1.12	1.11	1.11	1.10	1.10	1.03	1.03
Fixed operating cost, f ($¢ kWh^{-1})		0.80	0.70	0.72	0.71	0.51	0.40	0.30	0.28	0.34	0.37
Variable operating cost, w ($¢ kWh^{-1})		0.00	0.00	0.00	0.00	0.00	0.00	0.00	0.00	0.00	0.00
LCOE ($¢ kWh^{-1})		26.10	21.08	18.80	15.51	10.45	8.46	6.81	6.03	4.70	3.95

[a]2: 5 year MACRS DDB depreciation, 3: 20 year 150%-declining balance depreciation, 5: 100% bonus depreciation.
[1] Comello et al. (2020), [2] ABB (2020), [3] Steffen (2020), [4] Jordan et al. (2012), [5] Wiser and Bolinger (2016), [6] Tax Foundation (2020), [7] Tax Foundation (2012), [8] U.S. IRS (2019), [9] Bolinger et al. (2020), [10] Wiser et al. (2020)

are fixed operating cost for wind power plants. Fixed operating cost for solar PV facilities are calculated from data by Steffen et al. (2020). Annual average capacity factors of solar PV and wind energy facilities in Germany are calculated based on reported average capacity factors of individual plants in a particular year as provided by BMWi (2020) and IRENA (2020) for solar and wind, respectively.

Sensitivities

Tables 10–12 provide a comprehensive list of the annual input parameters and corresponding sources for the sensitivity calculations of the LCOE for solar, wind and PEM electrolyzers in Germany. We analyzed the impact of a more conservative WACC of 3% in 2019. Figs. 5–11 visualize the more conservative learning curves.

Funding We gratefully acknowledge financial support through the Deutsche Forschungsgemeinschaft (DFG, German Research Foundation) – Project-ID 403041268 – TRR 266, the Konrad-Adenauer Stiftung and the Joachim Herz Stiftung.

Acknowledgements Helpful comments were provided by Stefanie Burgahn, Stephen Comello, Nikolas Wölfing, and colleagues at the University of Mannheim and Stanford University. We also thank Yadira Funk, Lucas Politycki, and Philipp Scherer for providing valuable assistance with data collection and processing.

Table 6 Levelized cost dynamics for onshore wind in California

In 2019 $US	Source	2010	2011	2012	2013	2014	2015	2016	2017	2018	2019
Input Parameters											
Useful lifetime (years)	[1]	30	30	30	30	30	30	30	30	30	30
System price ($ kW^{-1})	[10]	2927	2805	2532	2382	2198	2000	2044	1959	1747	1678
Fixed operating cost ($ kW^{-1})	[2]	28.75	32.94	21.30	25.27	22.10	20.04	24.35	25.76	23.07	21.94
Variable operating cost ($¢ kWh^{-1})		0.00	0.00	0.00	0.00	0.00	0.00	0.00	0.00	0.00	0.00
Capacity utilization rate (%)	[2]	27.84	34.05	32.13	34.25	31.62	30.99	33.69	32.44	37.74	34.70
Cost of capital (%)	[3,10]	6.04	5.78	5.47	5.46	5.25	4.92	4.68	4.80	5.15	4.50
Degradation factor (%)	[5,10]	99.20	99.20	99.20	99.20	99.20	99.20	99.20	99.20	99.20	99.20
Investment tax credit (%)	[7]	0.00	0.00	0.00	0.00	0.00	0.00	0.00	0.00	0.00	0.00
ITC capitalization (%)	[1]	50.00	50.00	50.00	50.00	50.00	50.00	50.00	50.00	50.00	50.00
Production tax credit ($¢ kWh^{-1})		0.00	0.00	0.00	0.00	0.00	0.00	0.00	0.00	0.00	0.00
State tax rate (%)	[6]	8.84	8.84	8.84	8.84	8.84	8.84	8.84	8.84	8.84	8.84
Federal tax rate (%)	[7]	35.00	35.00	35.00	35.00	35.00	35.00	35.00	35.00	21.00	21.00
State tax depreciation method (–)[a]	[8]	3	3	3	3	3	3	3	3	3	3
Federal tax depreciation method (–)[a]	[8]	2	2	2	2	2	2	2	2	5	5
Levelized Cost											
Cost of capacity, c ($¢ kWh^{-1})		9.49	7.24	6.71	5.91	5.78	5.18	4.74	4.78	3.81	3.70
Tax factor, Δ (–)		1.13	1.12	1.12	1.12	1.11	1.11	1.10	1.10	1.03	1.03
Fixed operating cost, f ($¢ kWh^{-1})		1.28	1.20	0.82	0.92	0.87	0.80	0.90	0.99	0.76	0.79
Variable operating cost, w ($¢ kWh^{-1})		2.04	2.03	2.04	2.08	2.05	2.05	2.05	1.67	1.07	0.70

Table 6 (Continued)

LCOE ($¢ kWh^{-1}$)	11.96	9.32	8.31	7.51	7.29	6.53	6.12	6.27	4.70	4.61

[a]2: 5 year MACRS DDB depreciation, 3: 20 year 150%-declining balance depreciation, 5: 100% bonus depreciation.
[1] Comello et al. (2020), [2] ABB (2020), [3] Steffen (2020), [4] Jordan et al. (2012), [5] Wiser and Bolinger (2016), [6] Tax Foundation (2020), [7] Tax Foundation (2012), [8] U.S. IRS (2019), [9] Bolinger et al. (2020), [10] Wiser et al. (2020).

Table 7 Levelized cost dynamics for solar PV in Germany

In 2019 $US	Source	2010	2011	2012	2013	2014	2015	2016	2017	2018	2019
Input Parameters											
Useful lifetime (years)	[1]	30	30	30	30	30	30	30	30	30	30
System price ($ kW^{-1}$)	[2]	3705	2959	2341	2007	1600	1280	1162	1114	1113	899
Fixed operating cost ($ kW^{-1}$)	[3]	35.51	31.60	27.69	23.79	19.88	15.97	12.06	8.15	7.73	7.34
Variable operating cost ($¢ kWh^{-1}$)		0.00	0.00	0.00	0.00	0.00	0.00	0.00	0.00	0.00	0.00
Capacity utilization rate (%)	[4]	7.44	8.63	8.84	9.64	10.86	11.27	10.69	10.63	11.57	10.80
Cost of capital (%)	[5]	4.60	4.15	3.70	4.20	3.68	3.15	2.63	2.42	2.21	2.00
Degradation factor (%)	[1, 6, 7]	99.50	99.50	99.50	99.50	99.50	99.50	99.50	99.50	99.50	99.50
Federal tax rate (%)		30.00	30.00	30.00	30.00	30.00	30.00	30.00	30.00	30.00	30.00
Federal tax depreciation method (–)[a]	[8]	1	1	1	1	1	1	1	1	1	1
Levelized Cost											
Cost of capacity, c ($¢ kWh^{-1}$)		37.34	24.39	17.87	14.90	9.91	7.16	6.41	6.02	5.38	4.52
Tax factor, Δ (–)		1.15	1.14	1.13	1.14	1.13	1.11	1.10	1.09	1.09	1.08
Fixed operating cost, f ($¢ kWh^{-1}$)		5.76	4.42	3.79	2.98	2.22	1.72	1.37	0.93	0.81	0.83
Variable operating cost, w ($¢ kWh^{-1}$)		0.00	0.00	0.00	0.00	0.00	0.00	0.00	0.00	0.00	0.00
LCOE ($¢ kWh^{-1}$)		48.79	32.26	23.98	20.00	13.40	9.70	8.41	7.50	6.65	5.70

[a]1: 20 year linear depreciation schedule, 2: 16 year linear depreciation schedule.
[1] Comello et al. (2020), [2] IRENA (2020), [3] Steffen et al. (2020), [4] BMWi (2020), [5] Steffen (2020), [6] Jordan et al. (2012), [7] Wiser and Bolinger (2016), [8] BMF (2000)

Table 8 Levelized cost dynamics for onshore wind in Germany

In 2019 $US	Source	2010	2011	2012	2013	2014	2015	2016	2017	2018	2019
Input Parameters											
Useful lifetime (years)	[1]	30	30	30	30	30	30	30	30	30	30
System price ($ kW^{-1})	[2]	2271	2271	2063	2038	2011	1973	1936	1935	1904	1762
Fixed operating cost ($ kW^{-1})	[2]	73.00	73.00	68.00	68.00	64.00	64.00	56.00	53.63	51.25	48.88
Variable operating cost ($¢ kWh^{-1})		0.00	0.00	0.00	0.00	0.00	0.00	0.00	0.00	0.00	0.00
Capacity utilization rate (%)	[2]	24.00	24.00	24.00	24.10	25.30	26.10	26.90	27.10	31.30	31.10
Cost of capital (%)	[3]	4.60	4.70	3.90	3.90	4.00	3.30	2.70	2.47	2.23	2.00
Degradation factor (%)	[1, 4, 5]	99.50	99.50	99.50	99.50	99.50	99.50	99.50	99.50	99.50	99.50
Federal tax rate (%)		30.00	30.00	30.00	30.00	30.00	30.00	30.00	30.00	30.00	30.00
Federal tax depreciation method (–)[a]	[6]	2	2	2	2	2	2	2	2	2	2
Levelized Cost											
Cost of capacity, c ($¢ kWh^{-1})		7.09	7.17	5.94	5.84	5.56	4.86	4.29	4.13	3.41	3.08
Tax factor, Δ (–)		1.13	1.13	1.11	1.11	1.12	1.10	1.08	1.08	1.07	1.06
Fixed operating cost, f ($¢ kWh^{-1})		3.67	3.67	3.43	3.41	3.06	2.97	2.53	2.41	1.99	1.91
Variable operating cost, w ($¢ kWh^{-1})		0.00	0.00	0.00	0.00	0.00	0.00	0.00	0.00	0.00	0.00
LCOE ($¢ kWh^{-1})		11.68	11.79	10.04	9.92	9.26	8.31	7.18	6.85	5.65	5.19

[a] 1: 20 year linear depreciation schedule, 2: 16 year linear depreciation schedule.
[1] Comello et al. (2020), [2] IRENA (2020), [3] Steffen (2020), [4] Jordan et al. (2012), [5] Wiser and Bolinger (2016), [6] BMF (2000)

Table 9 Levelized cost dynamics for PEM electrolysis in Germany

In 2019 $US	Source	2010	2011	2012	2013	2014	2015	2016	2017	2018	2019
Input Parameters											
Useful lifetime (years)	[1]	25	25	25	25	25	25	25	25	25	25
System price ($\$\,kW^{-1}$)	[1]	2571	2295	2239	2807	2593	2643	1844	1572	1188	1064
Fixed operating cost ($\$\,kW^{-1}$)	[1]	77.13	68.86	67.18	84.22	77.78	79.30	55.32	47.17	35.64	31.91
Variable operating cost ($\$\,kg^{-1}$)	[1]	0.0019	0.0019	0.0019	0.0020	0.0020	0.0020	0.0021	0.0021	0.0021	0.0022
Conv. rate to hydrogen ($kg\,kWh^{-1}$)	[1, 2]	0.0166	0.0169	0.0172	0.0174	0.0177	0.0180	0.0183	0.0186	0.0189	0.0192
Avg. electr. buying price ($\$¢\,kWh^{-1}$)		6.20	6.95	5.72	5.00	4.28	4.06	3.66	4.20	5.29	4.42
Opt. capacity utilization rate (%)		97.20	98.72	95.00	96.31	97.97	98.49	93.50	88.70	72.51	75.84
Cost of capital (%)	[3]	4.60	4.70	3.90	3.90	4.00	3.30	2.70	2.47	2.23	2.00
Degradation factor (%)	[4]	98.40	98.40	98.40	98.40	98.40	98.40	98.40	98.40	98.40	98.40
Federal tax rate (%)		30.00	30.00	30.00	30.00	30.00	30.00	30.00	30.00	30.00	30.00
Federal tax depreciation method (–)[a]	[5]	2	2	2	2	2	2	2	2	2	2
Levelized Cost											
Cost of capacity, c ($\$\,kg^{-1}$)		1.44	1.26	1.16	1.41	1.27	1.18	0.80	0.69	0.62	0.51
Tax factor, Δ (–)		1.13	1.13	1.11	1.11	1.12	1.10	1.08	1.08	1.07	1.06
Fixed operating cost, f ($\$\,kg^{-1}$)		0.64	0.55	0.55	0.67	0.60	0.60	0.43	0.38	0.35	0.30
Variable operating cost, w ($\$\,kg^{-1}$)		3.77	4.20	3.30	2.87	2.47	2.33	1.99	2.12	2.45	2.10
LCOH ($\$\,kg^{-1}$)		6.04	6.18	5.14	5.11	4.49	4.23	3.29	3.25	3.46	2.93

[a]1: 20 year linear depreciation schedule, 2: 16 year linear depreciation schedule.
[1] Glenk and Reichelstein (2020), [2] IEA (2019), [3] Steffen (2020), [4] Buttler and Spliethoff (2018), [5] BMF (2000)

Table 10 Levelized cost dynamics for solar PV in Germany – 3% WACC

In 2019 $US	Source	2010	2011	2012	2013	2014	2015	2016	2017	2018	2019
Input Parameters											
Useful lifetime (years)	[1]	30	30	30	30	30	30	30	30	30	30
System price ($ kW^{-1})	[2]	3705	2959	2341	2007	1600	1280	1162	1114	1113	899
Fixed operating cost ($ kW^{-1})	[3]	35.51	31.60	27.69	23.79	19.88	15.97	12.06	8.15	7.73	7.34
Variable operating cost ($¢ kWh^{-1})		0.00	0.00	0.00	0.00	0.00	0.00	0.00	0.00	0.00	0.00
Capacity utilization rate (%)	[4]	7.44	8.63	8.84	9.64	10.86	11.27	10.69	10.63	11.57	10.80
Cost of capital (%)	[5]	4.60	4.15	3.70	4.20	3.68	3.54	3.41	3.27	3.16	3.00
Degradation factor (%)	[1, 6, 7]	99.50	99.50	99.50	99.50	99.50	99.50	99.50	99.50	99.50	99.50
Federal tax rate (%)		30.00	30.00	30.00	30.00	30.00	30.00	30.00	30.00	30.00	30.00
Federal tax depreciation method (–)[a]	[8]	1	1	1	1	1	1	1	1	1	1
Levelized Cost											
Cost of capacity, c ($¢ kWh^{-1})		37.34	24.39	17.87	14.90	9.91	7.16	6.41	6.02	5.38	4.52
Tax factor, Δ (–)		1.15	1.14	1.13	1.14	1.13	1.11	1.10	1.09	1.09	1.08
Fixed operating cost, f ($¢ kWh^{-1})		5.76	4.42	3.79	2.98	2.22	1.72	1.37	0.93	0.81	0.83
Variable operating cost, w ($¢ kWh^{-1})		0.00	0.00	0.00	0.00	0.00	0.00	0.00	0.00	0.00	0.00
LCOE ($¢ kWh^{-1})		48.79	32.26	23.98	20.00	13.40	10.20	9.30	8.42	7.60	6.50

[a] 1: 20 year linear depreciation schedule, 2: 16 year linear depreciation schedule.
[1] Comello et al. (2020), [2] IRENA (2020), [3] Steffen et al. (2020), [4] BMWi (2020), [5] sensitivity estimation, [6] Jordan et al. (2012), [7] Wiser and Bolinger (2016), [8] BMF (2000)

Table 11 Levelized cost dynamics for onshore wind in Germany – 3% WACC

In 2019 $US	Source	2010	2011	2012	2013	2014	2015	2016	2017	2018	2019
Input Parameters											
Useful lifetime (years)	[1]	30	30	30	30	30	30	30	30	30	30
System price ($ kW^{-1})	[2]	2271	2271	2063	2038	2011	1973	1936	1935	1904	1762
Fixed operating cost ($ kW^{-1})	[2]	73.00	73.00	68.00	68.00	64.00	64.00	56.00	53.63	51.25	48.88
Variable operating cost ($¢ kWh^{-1})		0.00	0.00	0.00	0.00	0.00	0.00	0.00	0.00	0.00	0.00
Capacity utilization rate (%)	[2]	24.00	24.00	24.00	24.10	25.30	26.10	26.90	27.10	31.30	31.10
Cost of capital (%)	[3]	4.60	4.70	3.90	3.90	4.00	3.30	3.23	3.15	3.08	3.00
Degradation factor (%)	[1, 4, 5]	99.50	99.50	99.50	99.50	99.50	99.50	99.50	99.50	99.50	99.50
Federal tax rate (%)		30.00	30.00	30.00	30.00	30.00	30.00	30.00	30.00	30.00	30.00
Federal tax depreciation method (–)[a]	[6]	2	2	2	2	2	2	2	2	2	2
Levelized Cost											
Cost of capacity, c ($¢ kWh^{-1})		7.09	7.17	5.94	5.84	5.56	4.86	4.29	4.13	3.41	3.08
Tax factor, Δ (–)		1.13	1.13	1.11	1.11	1.12	1.10	1.08	1.08	1.07	1.06
Fixed operating cost, f ($¢ kWh^{-1})		3.67	3.67	3.43	3.41	3.06	2.97	2.53	2.41	1.99	1.91
Variable operating cost, w ($¢ kWh^{-1})		0.00	0.00	0.00	0.00	0.00	0.00	0.00	0.00	0.00	0.00
LCOE ($¢ kWh^{-1})		11.68	11.79	10.04	9.92	9.26	8.31	7.55	7.33	6.14	5.70

[a] 1: 20 year linear depreciation schedule, 2: 16 year linear depreciation schedule.
[1] Comello et al. (2020), [2] IRENA (2020), [3] sensitivity estimation, [4] Jordan et al. (2012), [5] Wiser and Bolinger (2016), [6] BMF (2000)

Table 12 Levelized cost dynamics for PEM electrolysis in Germany – 3% WACC

In 2019 $US	Source	2010	2011	2012	2013	2014	2015	2016	2017	2018	2019
Input Parameters											
Useful lifetime (years)	[1]	25	25	25	25	25	25	25	25	25	25
System price ($ kW^{-1})	[1]	2571	2295	2239	2807	2593	2643	1844	1572	1188	1064
Fixed operating cost ($ kW^{-1})	[1]	77.13	68.86	67.18	84.22	77.78	79.30	55.32	47.17	35.64	31.91
Variable operating cost ($ kg^{-1})	[1]	0.0019	0.0019	0.0019	0.0020	0.0020	0.0020	0.0021	0.0021	0.0021	0.0022
Conv. rate to hydrogen (kg kWh^{-1})	[1, 2]	0.0166	0.0169	0.0172	0.0174	0.0177	0.0180	0.0183	0.0186	0.0189	0.0192
Avg. electr. buying price ($¢ kWh^{-1})		6.20	6.95	5.72	5.00	4.28	4.06	3.66	4.20	5.29	4.42
Opt. capacity utilization rate (%)		97.20	98.72	95.00	96.31	97.97	98.49	93.50	88.70	72.51	75.84
Cost of capital (%)	[3]	4.60	4.70	3.90	3.90	4.00	3.30	3.23	3.15	3.08	3.00
Degradation factor (%)	[4]	98.40	98.40	98.40	98.40	98.40	98.40	98.40	98.40	98.40	98.40
Federal tax rate (%)		30.00	30.00	30.00	30.00	30.00	30.00	30.00	30.00	30.00	30.00
Federal tax depreciation method (–)[a]	[5]	2	2	2	2	2	2	2	2	2	2
Levelized Cost											
Cost of capacity, c ($ kg^{-1})		1.44	1.26	1.16	1.41	1.27	1.18	0.84	0.74	0.65	0.54
Tax factor, Δ (–)		1.13	1.13	1.11	1.11	1.12	1.10	1.10	1.10	1.09	1.09
Fixed operating cost, f ($ kg^{-1})		0.64	0.55	0.55	0.67	0.60	0.60	0.43	0.38	0.34	0.28
Variable operating cost, w ($ kg^{-1})		3.77	4.20	3.30	2.87	2.47	2.33	1.99	2.13	2.48	2.13
LCOH ($ kg^{-1})		6.04	6.18	5.14	5.11	4.49	4.23	3.35	3.32	3.53	3.01

[a] 1: 20 year linear depreciation schedule, 2: 16 year linear depreciation schedule.

[1] Glenk and Reichelstein (2020), [2] IEA (2019), [3] sensitivity estimation, [4] Buttler and Spliethoff (2018), [5] BMF (2000)

Fig. 11 LCOE Sensitivity: Solar Power Germany

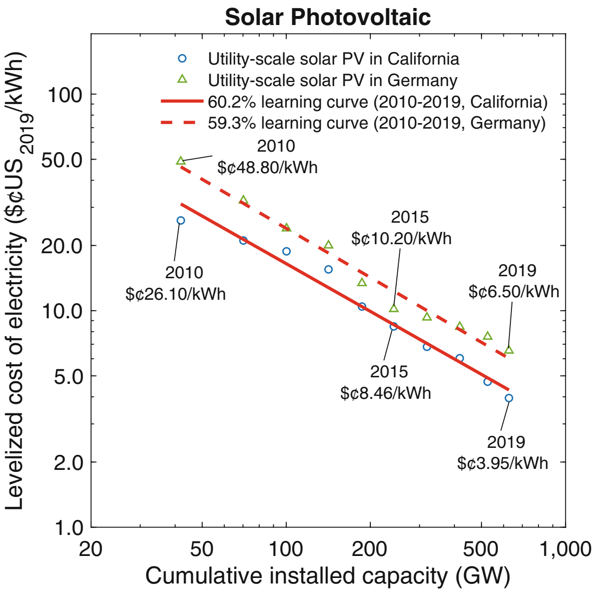

Fig. 12 LCOE Sensitivity: Wind Power Germany

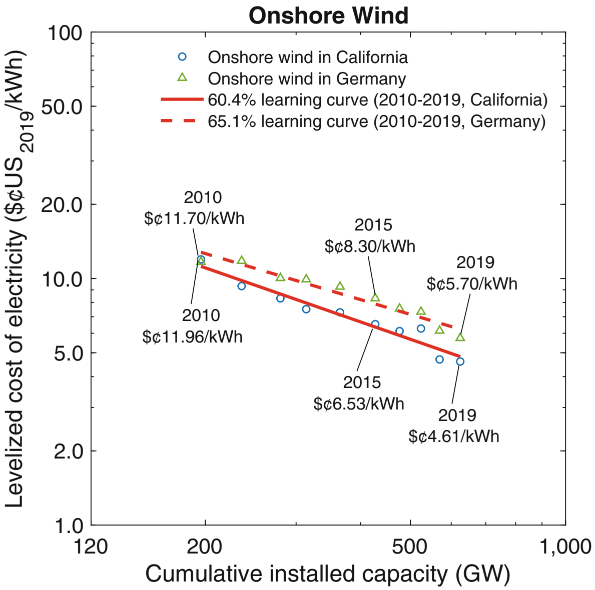

Fig. 13 LCOH Sensitivity: Germany

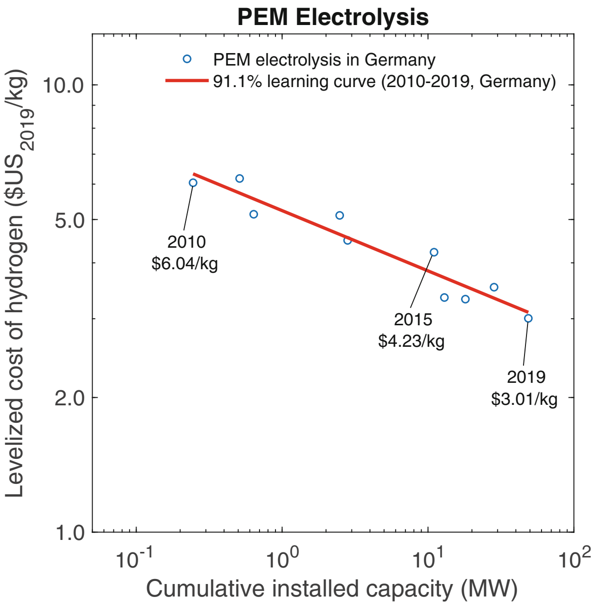

Supplement: Supplementary file 1 [file 41471_2021_114_MOESM1_ESM.pdf]
